# Critical left coronary main trunk stenosis, chronic occluded right coronary artery, left subclavian artery occlusion, severe aortic regurgitation and porcelain aorta in a patient with aortitis

**DOI:** 10.1186/s13019-020-01317-6

**Published:** 2020-09-25

**Authors:** Hideki Sasaki, Takashi Harada, Hiroshi Ishitoya, Osamu Sasaki

**Affiliations:** 1 Department of Cardiovascular Surgery, Ehime Prefectural Central Hospital, 83 Kasuga-cho, Matsuyama, Ehime 790-0024 Japan; 2 Division of Internal Medicine, Tokyo-Shinagawa Hospital, 6-3-22, Higashi-Oi, Shinagawa, Tokyo, 140-8522 Japan

**Keywords:** Left coronary main trunk stenosis, Porcelain aorta, Aortitis

## Abstract

**Background:**

Coronary involvement is rare but can be critical in patients with aortitis. Although cardiac ischemia can be resolved by coronary artery bypass grafting (CABG), patients complicated with cardiac ischemia, calcified aorta, and valve insufficiency pose difficult problems for surgeons.

**Case presentation:**

A 71-year-old woman was referred to our institution because of unstable angina. She had been previously diagnosed with aortitis and left subclavian artery occlusion. Contrast-enhanced computed tomography revealed severe left coronary main trunk stenosis, right coronary artery occlusion, and porcelain aorta. Ultrasonic echocardiogram showed severe aortic regurgitation. We performed emergent coronary artery bypass grafting, aortic valve replacement and ascending aorta replacement under hypothermic circulatory arrest.

**Conclusions:**

The technique of circumferential calcified intimal removal and reinforcement with felt strips was effective for secure anastomosis. Unilateral cerebral perfusion from the right subclavian artery enabled good visualization and sufficient time to perform distal anastomosis.

## Background

Coronary involvement is rarebit can be critical in patients with aortitis. Although cardiac ischemia can be resolved by coronary artery bypass grafting (CABG), patients complicated with cardiac ischemia, calcified aorta, and valve insufficiency pose difficult problems for surgeons. We present a 71-year-old woman with left main coronary trunk stenosis, occluded right coronary artery, left subclavian artery occlusion, severe aortic valve regurgitation and porcelain aorta with aortitis.

## Case presentation

**Fig. 1:**
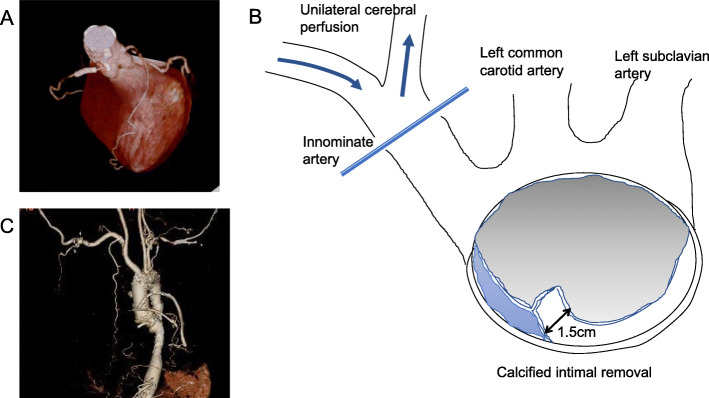
**a** Preoperative CT revealed 99% stenosis of left coronary main trunk and chronic occlusion of right coronary artery. **b** Circumferential removal of calcified intima during circulatory arrest. Unilateral cerebral perfusion via right subclavian artery was utilized. **c** Postoperative CT showed patent saphenous veins to left anterior descending artery and posterolateral branch

**Fig. 2:**
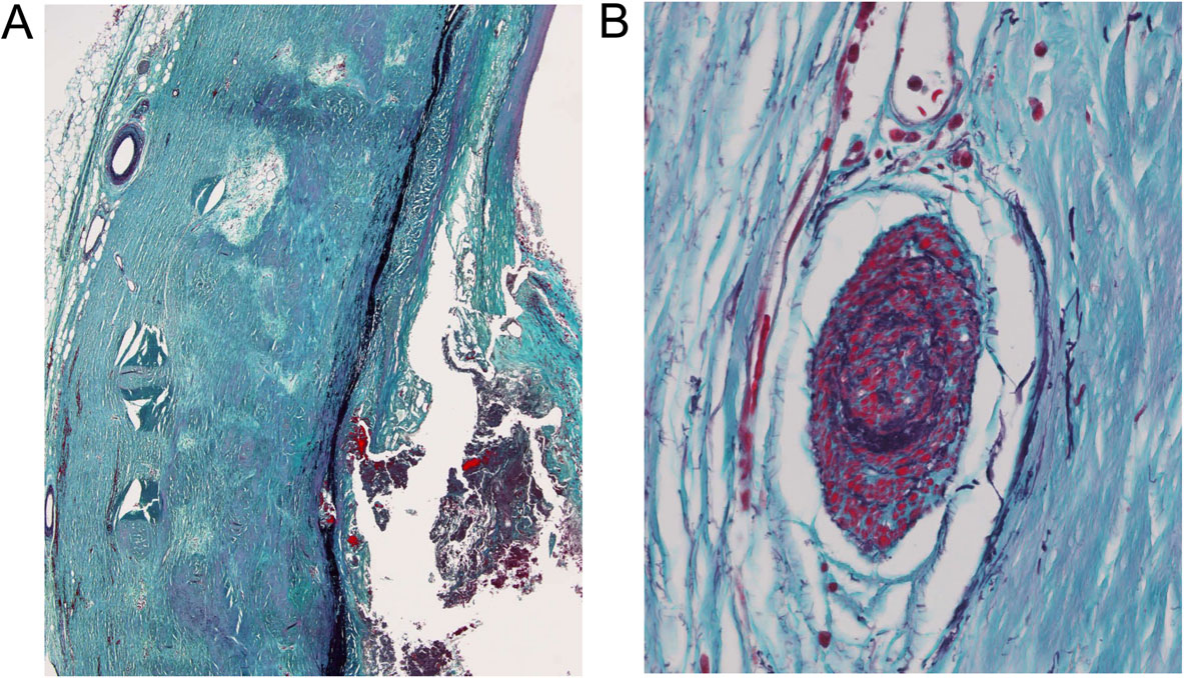
**a**. Histopathological examination revealed loss of medial elastic fibers, and hyalinized collagen fibers in the media and adventitia. **b**. Although perivascular monocyte infiltration was found, polynuclear giant cells were not found


A 71-year-old woman was transferred to our institution because of unstable angina. She had been previously diagnosed with aortitis and left subclavian artery (LSCA) occlusion. Her past medical history included hypertension and dyslipidemia. Twelve-lead electrocardiogram showed marked ST-segment depression in the precordial and inferior leads. Contrast-enhanced cardiac computed tomography (CT) revealed severe left coronary main trunk (LMT) stenosis, which had not been detected 9 months previously, right coronary artery (RCA) occlusion which had occurred previously (Fig. 1a), and porcelain aorta (Supplementary video). Ultrasonic echocardiogram showed severe aortic regurgitation and compromised left ventricular function (LVEF = 40%), which had been normal 9 months ago. Her chest discomfort worsened with time. We decided to perform emergent surgery. The right axillary artery (RAXA) and left femoral artery (LFA) were exposed. Saphenous vein grafts (SVGs) were harvested. Cardiopulmonary bypass (CPB) was established with RAXA/LFA perfusion and right atrial drainage. A retrograde cardioplegic cannula was inserted into the coronary sinus under transesophageal echocardiographic (TEE) guidance. A left ventricular venting cannula was inserted into the left ventricle (LV) through the right superior pulmonary vein. Systemic cooling was started. During cooling, the distal ends of SVGs were anastomosed to the posterolateral branch and left anterior descending artery, respectively on the beating heart. At an esophageal temperature of 23 °C, circulatory arrest was induced. Unilateral cerebral perfusion (UCP) from the RAXA was initiated (5 ml/kg). The calcified ascending aorta was incised. Antegrade cardioplegia was given from the SVGs. Retrograde cardioplegia was given from the coronary sinus as needed. The aorta was transected 1.5 cm proximal to the innominate artery. A 1.5 cm width of calcified intima was circumferentially removed from the media and adventitia. Teflon felt strips were applied inside and outside the aorta to create the distal anastomotic site (Fig. 1b). A 22 mm J Graft SHIELD NEO (JUNKEN MEDICAL, Co., Ltd., Tokyo, Japan) was anastomosed to the proximal aortic arch. Circulation was restored and the patient was rewarmed. The aortic valve was inspected. The NCC was redundant. The aortic valve was replaced with a 19 mm CROWN (LivaNova., London, United Kingdom). The proximal aorta was transected 1.5 cm distal to the sino-tubular junction. The same maneuver used for distal anastomosis was applied to create the proximal anastomotic site. The proximal ends of SVGs were anastomosed to the graft. Weaning from CPB was performed without difficulty with inotropic support. The postoperative course was uneventful. Contrast CT showed that all SVGs were patent (Fig. 1c). Histopathological examination revealed loss of medial elastic fibers, with replacement by hyalinized collagen fibers. Although monocyte infiltration was found in the perivascular area, polynuclear giant cells were not found (Fig. 2). These findings were compatible with the chronic phase of Takayasu’s arteritis. The patient was discharged home without any events on postoperative day 20. She has been followed in the outpatient clinic and her condition is thus far stable at 3 months.

**Additional file 1.**


## Discussion

Takayasu’s arteritis (TA) is a chronic, granulomatous vasculitis that causes stenosis, occlusion and ectasia of the aorta and its branches. Although coronary involvement is rare, it can be critical. Coronary ostial stenosis (COS) with normal distal part of the artery is a specific feature of TA.

In our case, the patient had not been in the active phase of TA for years. LMT stenosis progressively worsened over 9 months. It is not clear whether LMT stenosis in our patient was associated with TA, because stenosis occurred in the distal part of LMT, and not at the coronary ostium. In view of her diagnosis of dyslipidemia, this might have caused LMT stenosis over a short time period.

We had to perform emergent CABG because jeopardized left main coronary artery supplying the chronically occluded RCA caused global ischemia of the entire heart. Although percutaneous coronary intervention (PCI) for LMT stenosis can relieve ischemia quickly, it can be more dangerous compared with CABG because the RCA is already occluded and receives blood supply from the left coronary artery. Furthermore, PCI is not able to solve AR. Although the internal thoracic artery (ITA) is commonly used for CABG, it might not be adequate in patients with Takayasu’s arteritis because stenosis often occurs in the subclavian artery. However, the ITAs have been used in young patients in whom the subclavian arteries did not have stenosis [1, 2]. Although controversy exists, we believe that the SVG was an acceptable conduit in emergent surgery because her symptoms were unstable with deteriorated LV function. Although the LSCA was occluded, the right subclavian artery was not occluded in our patient. Although the right ITA was available, we did not use it. We thought that the right ITA over the prosthesis would make it difficult to perform maneuvers such as adding sutures to control bleeding from the graft-aorta anastomosis.

Another issue is calcified aorta, which is always a concern when surgeons try to clamp it. Transcatheter aortic valve insertion (TAVI) + off-pump CABG is the procedure of choice for patients with aortic valve stenosis, porcelain aorta and coronary ostial stenosis [3]. However, conventional AVR is required for patients with AR. In such cases, clamping the calcified aorta can cause embolization. Because calcification extended over the entire ascending aorta and proximal aortic arch in our case, we avoided clamping it and used hypothermic circulatory arrest. Takami et al. used short-term moderate hypothermic circulatory arrest for 3–4 min at a rectal temperature of 29 °C for internal inspection of the calcified aorta in patients requiring aortic/mitral valve surgery or coronary surgery. They performed debridement of calcification using a Cavitron Ultrasonic Surgical Aspirator (Tyco Healthcare, Mansfield, Mass) to apply safe crossclamping for selected patients [4]. This is applicable for some patients. However, pseudoaneurysm formation is another concern. Furthermore, we believe that the possibility of embolism is still higher than with graft replacement under circulatory arrest. On the other hand, any maneuver under direct vision using hypothermic circulatory arrest is accurate. We used a technique of circumferential calcified intimal removal and reinforcement with Teflon felt strips. The adventitia was thick and looked strong because TA might have been in the chronic phase. We made the anastomotic site using the thicker adventitia and Teflon felt strips both inside and outside the aorta. This technique contributed to avoiding bleeding from the suture holes.

Another issue is how to acquire good visualization of the proximal arch during hypothermic circulatory arrest. The proximal aortic arch was severely calcified, and its diameter was normal. Usual elasticity of the aorta was lost. It seemed difficult to obtain good visualization and enough time to apply the abovementioned technique using usual antegrade selective cerebral perfusion (ASCP) in which three perfusion cannulas are inserted into the innominate artery, left common carotid artery and LSCA, respectively. We considered that three cannulas in the calcified proximal aortic arch with normal size would prevent us from performing any maneuvers. Although controversy exists whether UCP can provide sufficient blood supply to both the left and right hemispheres compared to usual ASCP, we believe it is acceptable in emergent surgery [5]. In fact, in this hemodynamically unstable patient, we did not have enough time to perform head CT angiography or head magnetic resonance imaging/magnetic resonance angiography preoperatively. UCP provided us good visualization and enough time to perform distal anastomosis.

## Conclusion

Although the patient did not have active phase TA, LMT stenosis progressively worsened over a short period. The technique of circumferential calcified intimal removal and reinforcement with felt strips was effective for secure anastomosis and avoiding bleeding from the suture holes in the porcelain aorta. UCP from the RAXA provided good visualization and sufficient time to perform distal anastomosis.

## Data Availability

Not applicable.

## Abbreviations

CABG: Coronary artery bypass grafting
LSCA: Left subclavian artery
CT: Computed tomography
LMT: Left coronary main trunk
RCA: Right coronary artery,
RAXA: Right axillary artery
LFA: Left femoral artery
SVG: Saphenous vein graft
CPB: Cardiopulmonary bypass
TEE: Transesophageal echocardiography
LV: Left ventricle
UCP: Unilateral cerebral perfusion
TA: Takayasu’s arteritis
COS: Coronary ostial stenosis
PCI: Percutaneous coronary intervention
ITA: Internal thoracic artery
TAVI: Transcatheter aortic valve insertion
ASCP: Antegrade selective cerebral perfusion
